# On the power and promise of resonant diffraction for powders

**DOI:** 10.1063/4.0001042

**Published:** 2025-10-27

**Authors:** Kevin H. Stone, Sikhumbuzo M. Masina

**Affiliations:** SLAC National Accelerator Laboratory, Menlo Park, CA, USA

## Abstract

Significantly more information is available if X-ray scattering and spectroscopic techniques are combined. In crystallography, structural information is encoded in the intensity of Bragg peaks, with the complex scattering power of each atom given by f(Q,E) = f_0_(Q) + f'(E) + if''(E). Far from any resonant absorption edge, this scales as the atomic number, *Z*. The imaginary component, f'', gives rise to an energy dependent absorption, which displays a sharp edge jump at the resonant energy. The dispersive term, f', is a modification of the real part of the scattering power, effectively reducing the scattering strength for that atom in the vicinity of the resonant edge. The resonant terms f' and f'' and are related by a Kramers-Kronig transformation, such that the shape of one is sufficient to fully determine the other, as seen in Figure 1. This relationship also suggests that, if sufficient data can be collected for refinement of the energy dependent scattering factor for a specific crystallographic site, site specific XAS can be determined. It can be seen that the real part of the scattering factor shows a strong resonant effect even below the absorption edge. This is often exploited to change the scattering power in an element specific manner without inducing large absorption effects, which may increase beam damage to a sample or lead to complicated absorption corrections to the scattering intensity. Questions of elemental ordering within crystalline materials arise across a wide range of functional materials, from high-*T_c_ *superconducting materials such as YBa_2_Cu_3- x_M_x_O_7-δ_,[1] earth abundant photovoltaic absorber candidates such as Cu_2_Z_n_S_n_S_4_,[2] and lithium-rich layered oxide battery materials.[3] Cation ordering in such materials is often a function of processing conditions, making it important to study these in a form coinciding with their intended application as opposed to a more idealized system for fundamental studies. It is also common for the cation ordering to change, such as in lithium-rich layered oxide electrodes or above and below an order/disorder transition temperature either during material synthesis or under operating conditions. Realistic samples for use inspired science typically means that these materials must be studied in a polycrystalline form, making powder diffraction the preferred structural probe. While neutron diffraction, or co-refinement of X-ray and neutron diffraction data, is a proven means of differentiating between neighboring elements on the periodic table, neutron diffraction is not always suitable for the form factors (e.g. thin films), or time resolutions (e.g. rapid charging of Li-ion batteries) necessary for a given experiment. There also exist pathological cases where both X-rays and neutrons may provide insufficient scattering contrast.[4] In these cases, resonant X-ray diffraction can be used to enhance scattering contrast between otherwise similar elements. Complementary to structural studies from diffraction, X-ray absorption can provide critical information about the chemical state and local environment of an element within a sample. However, it is common in materials science to encounter samples comprised of a physical mixture of different phases. In such cases, the usual measurement approaches to X-ray absorption spectra will give only the weighted average of that physical mixture. Distinguishing these different phases with diffraction is generally straightforward, as the Bragg peaks which arise from different crystalline phases will not in general overlap, and any non-crystalline component will contribute to the diffuse background, only minimally impacting the observed peak intensities. Resonant diffraction then allows for phase- specific measurement of absorption spectra. This can be taken a step further to consider site-specific spectra within a single phase.[5] As different crystallographic sites contribute unequally to different Bragg peaks, the scattering factor of these different sites can be refined independently. This has been shown to be an effective way of distinguishing the spectral response of the same element at two distinct sites within a crystal structure. One can imagine then, isolating the chemical response of not just a particular element in a physical system, but also the response of that element from a specific phase or a specific crystallographic site within that phase. Powder diffraction is usually analyzed using a Rietveld refinement approach. The many factors affecting peak shapes, backgrounds, absorption corrections, and other influences of the experimental setup on the measured diffraction pattern have resulted in the adoption of a small number of Rietveld refinement programs which are widely used by the majority of the scientific community. This works extends those to allow for a Kramers-Kronig constrained refinement of the scattering factors on equal footing with other structural parameters typically refined from powder diffraction data. Effectively this results in a co- refinement of multiple diffraction patterns collected at multiple energies each including a refinable spectrum (or spectra in the case of distinct crystallographic sites or multiple phases containing the resonant element) of scattering factors. By imposing Kramers-Kronig consistency on the real and imaginary parts of these scattering factors and including any additional information, such as the averaged absorption spectra from a complimentary measurement, a self-consistent solution can be determined. This presentation will demonstrate the power of this approach and consider the demands and limitations of sample and data quality on the ultimate utility of this technique.
